# Networks for healthcare delivery: a systematic literature review

**DOI:** 10.1108/JHOM-09-2023-0262

**Published:** 2024-11-06

**Authors:** Ida Gremyr, Christian Colldén, Yommine Hjalmarsson, Marco Schirone, Andreas Hellström

**Affiliations:** Department of Technology Management and Economics, Chalmers University of Technology, Gothenburg, Sweden; Västra Götalandsregionen, Skövde, Sweden; Chalmers University of Technology, Gothenburg, Sweden

**Keywords:** Networks, Organisation, Configuration, Healthcare, Value, Service

## Abstract

**Purpose:**

Network configurations have been proposed as an efficient form of organisation and a promising area of research; however, a lack of conceptual clarity can be noted. The purpose of this review is to allow for a broad appreciation of network configurations and provide guidance for future studies of the concept.

**Design/methodology/approach:**

A systematic literature review was conducted based on the PRISMA method; Scopus, Web of Science, PubMed and the Cochrane Library were searched for conference proceedings and journal articles describing organisational networks to integrate resources aimed at care delivery. Around 80 articles were included in the final review and analysed thematically and by use of bibliographic coupling.

**Findings:**

The last decades have seen an increase in the frequency of articles describing networks for healthcare delivery. The most common contexts are care for multiple and/or long-term conditions. Three clusters of articles were found, corresponding to different conceptualisations of networks in healthcare: efficiency-enhancing cooperation, efficiency-enhancing integration and involvement for cocreation.

**Research limitations/implications:**

To increase conceptual clarity and allow the research on network configurations in healthcare to produce meta-learnings and guidance to practice, scholars are advised to provide ample descriptions of studied networks and relate them to established network classifications.

**Originality/value:**

The current review has only included articles including networks as a key concept, which provides a focused overview of the use of network configurations but limits the insights into similar approaches not described explicitly as networks.

**List of abbreviations**
CPY: citations per year

Set: Query
#7: #5 AND #4 AND #1
Refined by: LANGUAGES: (ENGLISH)
Indexes:  = CI-EXPANDED, SSCI, A&HCI, CPCI-S, CPCI-SSH, ESCI Timespan = All years
#6: #5 AND #4 AND #1
#5: TS=(organization OR organizations OR organisation OR organisations OR organised OR organized OR organizing OR organising OR organizational OR organizational OR administrat* OR management*)
#4: #3 OR #2
#3: TS=((“Resource integration”) OR (“Integration resources”) OR (“service network”) OR (“value network”))
#2: TS=(network* AND (co-creation OR co-production OR “value creation”))
#1: TOPIC: (health OR “health care” OR healthcare OR hospitals OR hospital)

Set: Query
#1: MeSH descriptor: [Health] explode all trees
#2: MeSH descriptor: [Delivery of Health Care] explode all trees
#3: MeSH descriptor: [Hospitals] explode all trees
#4: (health OR healthcare OR “health care” OR hospital*):ti,ab,kw (Word variations have been searched)
#5: #1 OR #2 OR #3 OR #4
#6: MeSH descriptor: [Models, Organizational] explode all trees
#7: MeSH descriptor: [Organization and Administration] this term only
#8: MeSH descriptor: [Health Facility Administration] this term only
#9: MeSH descriptor: [Hospital Administration] this term only
#10: (organization* OR organisation* OR organized OR organized OR organizing OR organizing OR administ* OR management*):ti,ab,kw (Word variations have been searched)
#11: #6 OR #7 OR #8 OR #9 OR #10
#12: (Network AND (co-creation OR co-production OR “value creation”)):ti,ab,kw (Word variations have been searched)
#13: (“Resource integration”):ti,ab,kw (Word variations have been searched)
#14: (“service network”):ti,ab,kw (Word variations have been searched)
#15: (“value network”):ti,ab,kw (Word variations have been searched)
#16: #12 OR #13 OR #14 OR #15
#17: #5 AND #11 AND #16

## Introduction

As increasing volumes of patients suffer from chronic conditions at the same time as both human and economic resources are scarcer, managers and scholars are looking for new ways to organise healthcare, e.g. turning to various improvement initiatives (Colldén *et al.*, 2017). Stabell and Fjeldstad (1998) developed a framework that started with a process-oriented perspective but proposed additional approaches to further understand value creation in an organization, where the *chain* (process) was complemented with *shop* and *network*. First, in terms of organisation healthcare is traditionally organised based on a *shop configuration*, where resources (for example, different medical professionals) are allocated around a unique problem, such as a specific diagnosis (Fjeldstad *et al.*, 2019; Hwang and Christensen, 2008). Second, in attempts to improve efficiency of care delivery, streamlining in *chain configurations* has been advocated, sometimes connected to improvement concepts like lean healthcare (de Souza, 2009). Such attempts have brought some efficiency gains for care providers, but have often been narrowly applied to achieve improvements within one department, rather than for a system (Mazzocato *et al.*, 2010). Third, contemporary healthcare systems often suffer from fragmentation, causing inefficiencies and poor care quality (Frandsen *et al.*, 2015). To integrate care and improve cost-efficiency, value creation by *network configurations* has been promoted (Christensen *et al.*, 2009; Fjeldstad *et al.*, 2019) and increasingly used (Addicott and Ferlie, 2007). Moreover, networks can be used both as a general approach for how to organise healthcare systems (Angiola and Bianchi, 2017; Fleury, 2005; Parchman *et al.*, 2011) or applied specifically for a certain medical or social context. For example, specific models have been described for mental healthcare (Alvarado *et al.*, 2012; Fleury *et al.*, 2008; Væggemose *et al.*, 2018), trauma care (Bazzoli *et al.*, 1998), cancer (Eriksson *et al.*, 2020), and people ageing with HIV (Siegler and Brennan-Ing, 2017).

For healthcare, networked solutions have been suggested under various labels, such as *service networks* (Black and Gallan, 2015; Tzannis, 2013), *service supply networks* (Sampson *et al.*, 2015), *integrated service networks* (Fleury, 2006), *service delivery networks* (Spurrell *et al.*, 2016; Tax *et al.*, 2013), and *integrated healthcare networks* (Vargas *et al.*, 2015). The solutions generally refer to formal or informal integration of various resources needed in a care process (Eriksson and Hellstrom, 2021; Fleury, 2006). Spurrell *et al.* (2016) proposed a holistic perspective on service networks, emphasizing value creation in the collaborative space between the patient's personal network, the healthcare provider network, and other stakeholders' networks. This approach aligns with the ecosystem viewpoint found in service-dominant logics literature and acknowledges co-creation mechanisms at the microsystems level (Eriksson and Hellstrom, 2021). Co-creation is emphasised as patients being collaborators to staff (Nordgren, 2008), but also with focus on creating a safe collaboration climate among staff (Mannion *et al.*, 2023). In this review, we embrace various conceptualizations and aim to explore how network configurations have been utilized, regardless of specific terminology.

In addition to the claim for improved efficiency, there are several other rationales for the use of network configurations. First, integration of care in networks can reduce fragmentation and improve continuity of care for patients (Abba-Aji *et al.*, 2019; Lorant *et al.*, 2019). Second, networks have been suggested to improve clinical outcomes (Alvarado *et al.*, 2012; Fleury, 2006; Joseph, 2006), even though such results are sometimes difficult to realise (Lorant *et al.*, 2019; Moore *et al.*, 2007). Some key aspects in realising the benefits suggested, and anticipated, relates to, e.g. culture, interorganisational collaboration and leadership (Bhat *et al.*, 2022), where good leadership is one aspect that characterised networks with positive impact on quality of care (Brown *et al.*, 2016). Third, the use of networks has been argued to be particularly relevant in care for chronic conditions (Angiola and Bianchi, 2017; Collden *et al.*, 2021; Joseph, 2006; Tzannis, 2013) and multimorbidity (Breton *et al.*, 2017; Sampson *et al.*, 2015; Siegler and Brennan-Ing, 2017), where multiple providers and stakeholders need to be involved over extended periods of time. Fourth, networks also have the potential to improve access to care for populations that are otherwise hard to reach. For example, they have been used to improve care access in rural areas (Arpiainen and Lilius, 2020; Coburn, 2001; Ickenstein *et al.*, 2005; Jackson *et al.*, 2019) and reach homeless persons (Morrissey *et al.*, 1997, 2002). A fifth rationale for the use of networks is to promote co-creation of care, empowering patients to participate more actively in their own care (Barrett *et al.*, 2014; Eriksson and Hellstrom, 2021), and enhance patient experiences (Schiavone, 2020).

Networks can take on various forms; at a system level, Angiola and Bianchi (2017) distinguished between *patient-governed networks*, *lead-organisation-governed networks*, and *network administrative organisations,* in which a separate institution serves as a macro-level facilitator of network cooperation. Other scholars have attempted to describe the level of integration of networks. For example, Provan *et al.* (2009) operationalised an organisation’s involvement in a network as its “centrality” and showed that a higher degree of centrality is related to positive outcomes, while Leutz (1999) distinguished among three levels of integration: linkage, coordination, and full integration. Thus, the idea of using healthcare networks for improving healthcare delivery has gained traction in various fields. However, there is a lack of conceptual clarity due to different labels and varying degrees of integration among network actors. To address this, we conduct a literature review on network organization in healthcare guided by three research questions:

RQ1. What are the rationales for using network configurations in healthcare?

RQ2. In what contexts are network configurations used?

RQ3. How are networks operationalised in healthcare in terms of actors and their interrelations?

In relation to existing literature the envisioned contributions are to provide an integrative classification that does not mainly focus on specific aspects like network governance (Provan and Kenis, 2008) or a specific medical condition (e.g. Bazzoli *et al.*, 1998). In addition, as network configurations in healthcare is a phenomenon addressed in various fields a systematic literature review as a method contributes to academics by providing a synthesis based on methodological rigour and to professionals by pointing to, and synthesising, knowledge from a broad range of areas (Tranfield *et al.*, 2003).

## Method

Motivated by the perceived diversity of earlier literature on the phenomenon of networks in healthcare, this study builds on a systematic literature review (Tranfield *et al.*, 2003).

### Review process

**Figure 1:**
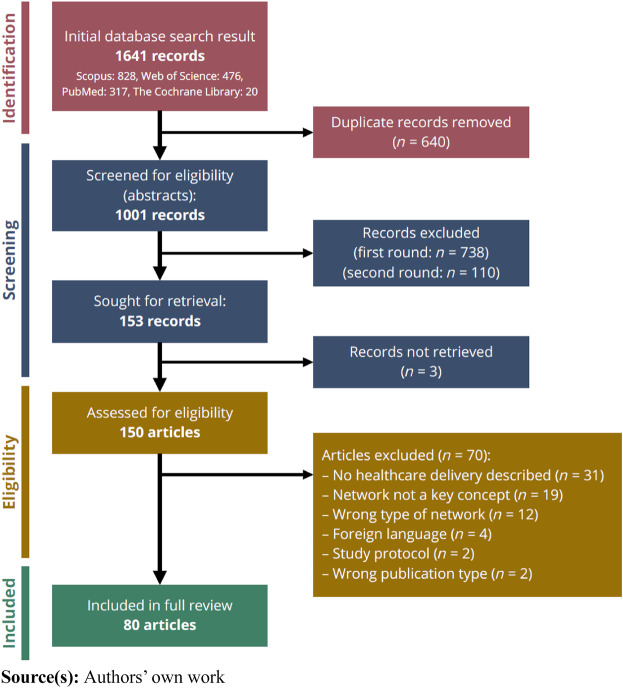
PRISMA flowchart

The systematic literature review was conducted in line with the PRISMA flow diagram (Moher *et al.*, 2009) (Figure 1). The PRISMA method was applied (Torraco, 2016). First, the research questions were outlined and guided the design of a search strategy based on the PICo framework (Miller and Forrest, 2001), with a focus on capturing organisational networks to integrate resources in healthcare. Since the phenomenon spans several fields, four databases were selected for the search: Scopus, Web of Science, PubMed, and The Cochrane Library. The search strategy (Appendix) was adapted to the different databases regarding search fields and index terms. There were no restrictions on publication type or date, but non-English publications were excluded. In total, the search rendered 1,641 records, including 640 duplicates.

Second, titles and abstracts of the remaining 1,001 records were screened. In this step, journal articles and conference proceedings were included. Master’s and doctoral theses were not included as a publication type, due to challenges with access and variation in peer review and assessment practices. Abstracts were included if organisational networks and/or resource integration were key concepts, and the context was healthcare. Abstracts relating to pure IT networks and microsystem networks (that is, the network around individual patients) were excluded. In a first round, 738 abstracts were excluded as they did not meet the inclusion criteria (see Box 1 for a full list of inclusion criteria). Next, the authors compared undecided abstracts, resulting in two additional criteria for inclusion: the articles should contain a *description* of one or several networks, and the purpose of the network should be *healthcare delivery* (excluding, e.g. networks for research and innovation). In a second round of screening 110 additional records were excluded.

Box 1 Inclusion criteria for the screening of abstracts and titles.

(All to be met for inclusion)

- Full paper available in English
- Journal paper or conference proceedings
- Networks used as a central concept in the article
- Network refers to organisation (e.g. IT-networks or personal networks excluded)
- Include a description of one or several actual networks in healthcare context (empirical or conceptual but concrete or planned)
- Purpose of the network is care delivery, not only research or innovation networks
- Healthcare used as the – or one of the – key contexts

Third, the remaining records were reviewed in full text; 150 were obtained and assessed for eligibility. Again, the authors shared the task of assessing the articles, switching parts of the body of records so that each author had assessed all the finally included records during the process. In the full-text review, 70 additional articles were excluded. Finally, 80 articles were included in the review.

### Thematic analysis

A thematic analysis (Brooks *et al.*, 2015) was conducted, starting with preliminary coding of the data based on *a priori* themes linked to the research questions. Next, all authors coded a sample of articles to test and revise the initial coding template and identify emerging subthemes. Definitions of categories and subthemes were discussed jointly to establish a final coding template with seven categories, hence being a combination of *a priori* and inductively derived codes:

1. Empirical or conceptual article
2. Rationale for the use of networks
3. Care context (medical speciality or patient group)
4. Methods used in the study
5. Types of actors involved in the described network
6. Types of connections between network actors (from loose cooperation to strictly co-organised)
7. Types of outcomes described

### Bibliometric analysis

Bibliographic coupling, a method for mapping scientific literature, groups documents based on shared references (Kessler, 1963). Publications with many common references are assumed to cover similar topics. Metadata from Scopus and Web of Science Core Collection was extracted for this review. Data from 75 documents in Scopus and 4 in Web of Science Core Collection were imported into R version 4.2.0 (https://www.r-project.org). The bibliometrix package, designed for bibliometric mapping, was used to calculate coupling relations and create a network map (Aria and Cuccurullo, 2017). Additionally, it provided descriptive statistics, such as the most cited journals.

The visualisation was created in four steps. First, once the data were imported from the two databases, they were converted to R data frames and a data-cleaning routine was performed. Second, the 2 R data frames were merged into a dataset comprising 79 documents. Third, the relations of bibliographic coupling were calculated and graphically transformed into links connecting the documents in the dataset on a visualisation map. Forth, documents were aggregated into clusters based on the bibliographic coupling links.

**Figure 4:**
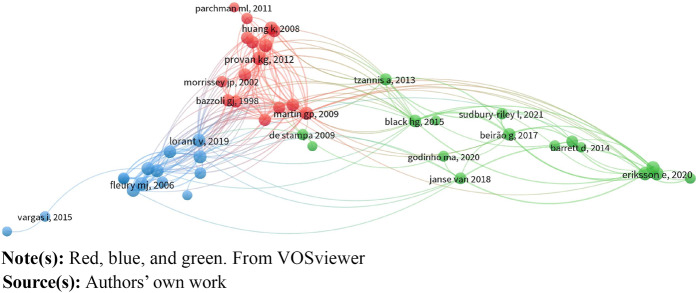
Visualisation of the three bibliographically coupled clusters

Bibliometrix has a notable feature – it's compatible with VOSviewer, an advanced bibliometric mapping tool developed by the University of Leiden (van Eck and Waltman, 2010). This compatibility allows for exporting cleaned and analysed data from R to VOSviewer. To enhance readability, documents without at least one bibliographic coupling relation were excluded from the VOSviewer map. Three clusters emerged based on bibliographic couplings, these were analysed using thematic coding, with each cluster containing a minimum of 10 documents for meaningful insights. The LinLog layout technique in VOSviewer was employed to enhance map graphics (van Eck and Waltman, 2022). Figure 4 displays the bibliographic coupling network, and Table 3 provides detailed cluster information.

**Table 3:** Characteristics of the three clusters

Cluster	Description	Time range	Included articles
1 (red): *efficiency-enhancing cooperation*	Networks used for efficiency gains, mostly through cooperation between and within healthcare providers. Dominated by quantitative studies published in healthcare journals	1997–2019	Angiola and Bianchi (2017), Bazzoli *et al.* (1998), Brewster *et al.* (2019), Huang (2014), Huang *et al.* (2019), Huang and Provan (2007, 2008), Provan (2006), Lam and Li (2020), Martin *et al.* (2009), Matinheikki *et al.* (2017), Moore *et al.* (2007), Morrissey *et al.* (1997, 2002), Parchman *et al.* (2011), Provan and Huang (2012)
2 (blue): *efficiency-enhancing integration*	Increased efficiency and decreased fragmentation are common rationales. Networks usually include both healthcare and social service actors, often co-organised or integrated by government facilitated cooperation. Mixed journals and methods but often involving psychiatry	2002–2021	Alvarado *et al.* (2012), Bohnet-Joschko *et al. *(2019), Breton *et al.* (2017), Fleury (2006), Fleury *et al.* (2008, 2017), Fleury and Mercier (2002), Fleury *et al.* (2002), Longpre and Dubois (2015), Lorant *et al.* (2016, 2019), Nicaise *et al.* (2021), Siegler and Brennan-Ing (2017), Vargas *et al.* (2015)
3 (green): *involvement for co-creation*	Mixed rationales, but the only cluster focusing on co-creation. Patients and families are often explicitly involved in addition to healthcare and social service actors. Mixed contexts, but less often psychiatry than in the other clusters. Mainly qualitative case studies published in journals from different fields but more often service-oriented journals compared to other clusters	2006–2021	Barrett *et al.* (2014), Beirão *et al. *(2017), Black and Gallan (2015), De Stampa *et al.* (2009), Eriksson *et al.* (2020, 2021), Eriksson and Hellstrom (2021), Fjeldstad *et al.* (2019), Godinho *et al.* (2020), Janse van Rensburg *et al.* (2018), Joseph (2006), Lantos and Simon (2018), Sampson *et al.* (2015), Sudbury-Riley and Hunter-Jones (2021), Tzannis (2013), Væggemose *et al.* (2018)

**Source(s):** Authors’ own work

## Results

Initially, a descriptive analysis is presented highlighting key aspects such as common journals, authors, and study contexts. Subsequently, the bibliometric analysis outlines three distinct healthcare network configurations.

### Descriptive analysis

**Figure 2:**
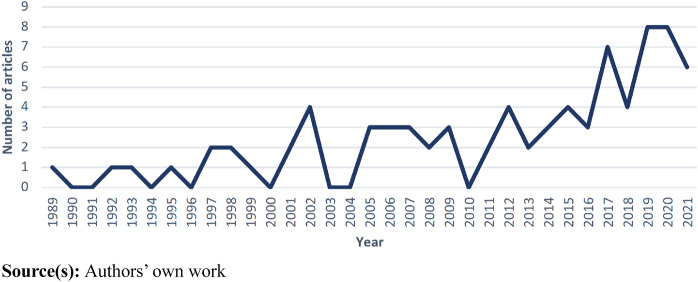
Trend of published articles in the period 1989–2021

The first article included in the review was published in 1989 and a total of eight articles were published during the 1990s. From the beginning of the new millennium, a slightly positive trend is noticed in the frequency of network descriptions in published articles, up until 2017 when a sharper increase can be observed, as shown in Figure 2. The highest number of included articles, eight per year, was noticed in 2019 and 2020.

The 80 articles reviewed were published in 60 different journals, most included only one article. Table 1 presents the 11 journals that published more than one of the articles included in full-text review. Most of the journals in Table 1 focus on both management and healthcare; for example, public administration and health service management.

**Table 1:** Top journals based on number of published articles, their total number of citations, and CPY

Journal	Number of articles	Citations	CPY (total)
*BMC Health Services Research*	5	68	5.2
*Journal of Public Administration Research and Theory*	3	235	16.8
*International Journal of Integrated Care*	3	36	7.2
*Public Management Review*	2	81	5.4
*Industrial Marketing Management*	2	43	4.8
*Health Service Management Research*	2	47	2.2
*International Journal of Health Planning and Management*	3	29	1.5
*British Journal of Management*	2	14	14.0
*Health and Social Care in the Community*	2	16	1.1
*Public Administration Review*	2	73	7.3
*Health Care Management Review*	2	13	0.5
*Social Science and Medicine*	2	20	0.8
*Psychiatric Services*	2	98	3.9

**Note(s):** The number of citations is based on the complete citation index of the bibliometric databases used for the analysis, *Web of Science Core Collection* (Clarivate Analytics) and *Scopus* (Elsevier). CPY (citations per year) is the yearly average number of times each journal (that is, the articles, out of the 79 documents included in the dataset, published in that specific journal) has been cited

**Source(s):** Authors’ own work

While the articles reviewed are published in many different journals, when looking at scholarly recognition some articles stand out as more influential. The 10 most cited articles are presented in Table 2.

**Table 2:** The 10 most cited articles

Author	Year	Title	Citations	CPY
Beiro, G. *et al.*	2017	Value cocreation in service ecosystems	115	19.17
Provan, K. *et al.*	2009	The evolution of structural embeddedness and organisational social outcomes in a centrally governed health and human services network	109	7.79
Black, H. and Gallan, A.	2015	Transformative service networks: cocreated value as well-being	86	10.75
Martin, G. *et al.*	2009	Leadership, service reform, and public-service networks: the case of cancer-genetics pilots in the English NHS	81	5.79
Huang, K.	2007	Structural embeddedness and organisational social outcomes in a centrally governed mental health services network	57	3.56
Matinheikki, J. *et al.*	2017	New value creation in business networks: The role of collective action in constructing system-level goals	35	5.83
Huang, K.	2014	Knowledge sharing in a third party-governed health and human services network	35	3.89
Archbald-Pannone, L. *et al.*	2020	COVID-19 collaborative model for an academic hospital and long-term care facilities	26	8.67
Eriksson, E. *et al.*	2020	Collaborative public management: coordinated value propositions among public service organizations	24	8.00

**Note(s):** The number of citations is based on the complete citation index of the bibliometric databases used for the analysis, *Web of Science Core Collection* (Clarivate Analytics) and *Scopus* (Elsevier). CPY (citations per year) is the yearly average number of times each article has been cited and the 10 articles with the highest numbers are included in the table

**Source(s):** Authors’ own work

**Figure 3:**
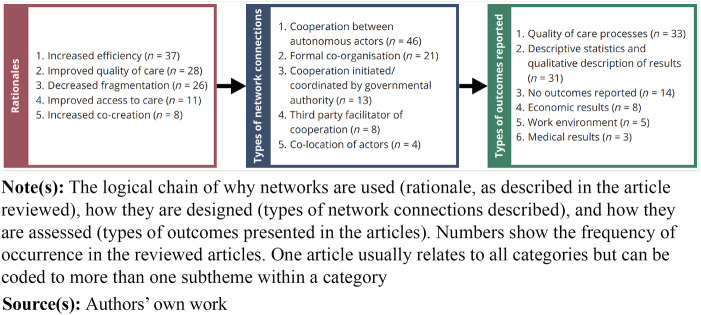
Rationales, designs, and assessments of networks

Below, the articles reviewed will be described in terms of the accounted rationales for a network configuration, the types of connections in the network, and the types of outcomes reported, as summarised in Figure 3.

In terms of rationales or drivers to organise in networks the articles reported, in descending order of frequency, increased efficiency (*n* = 37), improved quality of care (*n* = 28), decreased fragmentation (*n* = 26), improved access to care (*n* = 11), and the opportunity for patients and other stakeholders to take part in value co-creation (*n* = 8). The most common context in which networks were used was mental healthcare (*n* = 30), followed by care for elderly (*n* = 16). While many other patient groups are represented, a common theme is that a vast majority concern chronic, or long-term care. In addition to healthcare providers, the actors involved in the networks were mainly municipal and social services (*n* = 38). Governmental authorities and payers were included in 14 networks, while the explicit inclusion of patients (*n* = 8) and families (*n* = 7) were rarer.

The degrees to which the networks were formalised were not always clearly described, some articles studied the connections and interactions within the networks in detail, others only superficially mentioned included actors. However, the review points to non-formalised cooperation between autonomous actors as the most common type of network connections (*n* = 46). Formally co-organised networks were the next most common type (*n* = 21), followed by networks with a governmental authority initiating and coordinating the network (*n* = 13). Network connections directly facilitated by a separate third party were unusual (*n* = 8), and in these cases the facilitating actors were often governmental authorities, thus overlapping the government-led networks. Co-location of actors was reported only in four cases.

In terms of reported outcomes, assessments and evaluations of medical results were rare (*n* = 2), while 32 articles reported outcomes in terms of improved quality of the care processes. Almost as many (*n* = 31) only presented descriptive statistics of the outcome of healthcare networks, and 11 of the articles did not present any measurable outcomes. This may be related to study type, as the most common study type was case studies (43 of the 80 articles), although both qualitative and quantitative methods were used to similar extents.

### Three approaches to network configurations in healthcare

The bibliometric analysis resulted in three clusters of articles, referred to as *efficiency-enhancing cooperation*, *efficiency-enhancing integration*, and *involvement for cocreation*. The clusters are visualised in Figure 4 and their characteristics are outlined in Table 3.

### Cluster 1: efficiency-enhancing cooperation

The first cluster is characterised by networks consisting of cooperating healthcare providers, such as hospitals and primary care organisations. The included actors sometimes belong to the same provider organisation (Parchman *et al.*, 2011), and sometimes to different organisations providing care for the same conditions but in different geographical areas, or different parts of the care process (Angiola and Bianchi, 2017; Matinheikki *et al.*, 2017). A recurring purpose is to facilitate cooperation between health and social care (Brewster *et al.*, 2019; Moore *et al.*, 2007), municipal and specialised care (Angiola and Bianchi, 2017), and for-profit and public or non-profit organisations (Lam and Li, 2020; Provan and Huang, 2012). The most common rationale for use of networked organisation is to improve efficiency, but some papers also mentioned decreased fragmentation. The contexts are dominated by mental health (e.g. Huang, 2014; Morrissey *et al.*, 2002) and care for elderly (e.g. Angiola and Bianchi, 2017; Lam and Li, 2020), and the studies are predominantly quantitative.

### Cluster 2: efficiency-enhancing integration

In the second cluster, integration of care (that is, decreased fragmentation) and efficiency improvements are both common rationales for using a network configuration. Concerning contexts, this second cluster is the one that is most dominated by mental healthcare of the three (10 of 14 articles) and most networks include both healthcare and social service actors. Cooperation between different independent actors is common, as in all clusters, but co-organised (e.g. Breton *et al.*, 2017) and other formalised and centrally controlled networks (for example, government-controlled networks (Fleury and Mercier, 2002; Fleury *et al.*, 2002; Vargas *et al.*, 2015)) tend to be more common in this cluster than in the other ones. Hence, the cluster can be characterised as including networks with a higher level of integration between the included actors, than Cluster 1. The study designs are mixed, with both qualitative and quantitative methods and often case descriptions. A typical example from this cluster is Lorant *et al.* (2019) who studied the effectiveness of networks of health and social services for severely mentally ill patients in Belgium in a case-control study.

### Cluster 3: involvement for co-creation

The third cluster is dominated by qualitative case studies in diverse contexts. Mental health occurs, but only in a few articles. Instead, contexts often include chronic and/or complex care. For example, Sampson *et al.* (2015) studied patients with comorbidities who require care from multiple networked healthcare providers. Furthermore, healthcare and social service actors are common network components, but this cluster stands out because it includes families and patients as acknowledged parts of networks, a trait that is found only in this cluster. This is also the only cluster where co-creation is pronounced as a rationale for the use of network configurations. For example, Eriksson and Hellström (2021) studied the integration of resources from the personal sphere, public sector, private sector, and third sector with the patient’s own resources, in the contexts of cancer screening and rehabilitation. Hence, this cluster can be characterised as focusing on a level closer to the actual healthcare delivery than the other two, which generally apply a macro perspective. There are also several examples of digital solutions to facilitate network interactions in this cluster; for example, Godinho *et al.* (2020), who described “community health alliances” that utilise digital health solutions to engage citizens and deliver integrated care.

## Discussion

Networks have been applied and discussed in many care contexts, but the most common contexts are mental healthcare and care for elderly. A common characteristic of these medical areas is that care is needed over extended periods of time and that the medical conditions often require not only medical treatment, but also social support. Hence, multiple actors are inherently required in the care of the individual patient and their collaborations and organisational culture is central (Mannion *et al.*, 2023). Thus, this care corresponds to the fundamental properties of networks (Stabell and Fjeldstad, 1998). Other types of long-term care contexts are also found in the material, such as diabetes, chronic obstructive pulmonary disease, ischemic heart disease (Joseph, 2006) and more general approaches to support chronically ill patients through primary care (Angiola and Bianchi, 2017; Tzannis, 2013). Similarly, patients who have comorbidities have been shown to benefit from network configurations (Sampson *et al.*, 2015).

The identified approaches to networks all aim to improve integration and efficiency. However, there are also notable differences. First, there is a diversity in perspectives from which the networks are described. Common cross the first two clusters are that networks are described from a provider perspective. That is, organisations that provide care for patients (usually healthcare or social care) form networks to improve their services for the gain of the organisations and/or the patients. Customer-initiated coordination is found less frequently, but could be related to cluster 3 with a focus on individual patients and other individual actors in a network.

Second, the review points to diversity of actors involved in the networks. Angiola and Bianchi (2017) recognised the importance of the level of individuals within networks, arguing that “the implementation of ‘rational/technocratic’ factors is important but not sufficient to enhance collaboration [but that] integration at the ‘professional level’ should be kept in mind [and that] the role of network (case) managers is paramount” (p. 575). Similarly, Nicaise *et al.* (2021) showed that the quality of collaboration in service networks is dependent on an appropriate balance between interpersonal and interorganisational mechanisms, for fragmentation to decrease. At a micro level, Væggemose *et al.* (2018) also showed that different logics are applied by public service officials and civil society volunteers, and these different logics need to be integrated for co-creation of care to take place. Taken together, the relations and interactions between teams and individuals within organisational networks can be of similar importance for the network to serve its purpose; whether it is used to increase efficiency or quality-of-care, reduce fragmentation, or empower patients to co-create value. Cluster 3 highlights the importance of micro-level involvement of actors in different types of networks to cocreate care in an ecosystem of individual and organisational actors (Grönroos and Gummerus, 2014). The networks described in this cluster correspond to different modes (Provan and Kenis, 2008), with examples of co-organised services (Joseph, 2006), collaborations between independent actors (Lantos and Simon, 2018), and services facilitating networked interactions digitally (Godinho *et al.*, 2020). Notably, three of the four articles with the highest CPY are included in Cluster 3, and of the remaining seven articles on the top 10 CPY list, six are included in Cluster 1 (published 2007–2014) and none in Cluster 2. While these results are only indicative, they may suggest a trend towards a greater interest in micro-level interactions in networks.

Third, there is a diversity in terms of the degree of integration between actors in the networks. A difference between clusters 1 and 2 lies precisely in the degree of integration between the included actors (Leutz, 1999; Provan *et al.*, 2009). In Cluster 1, networks rely primarily on mutual coordination between actors, while Cluster 2 includes descriptions of networks that are closer to full integration (Leutz, 1999). In relation to the three modes of networks described by Provan and Kenis (2008) – lead organisation-governed networks, participant-governed networks, and network administrative organisations – Cluster 1 can be seen to correspond to participant-governed networks, while Cluster 2 corresponds more to lead organisation-governed networks and network-administrative organisations. These two clusters overlap in terms of network modes and degree of integration, but the findings support the relevance in making the levels of integration in different networks explicit. Relating to modes of networks (Provan and Kenis, 2008) or measuring the “centralisation” of networks (Brewster *et al.*, 2019; Huang and Provan, 2007; Provan *et al.*, 2009) are examples of ways to make this property explicit, which can help to gain a more fine-grained understanding of how networks can be designed.

### Agenda for future research

The literature reviewed does not seem to emanate from one or a few central references. Instead, it seems to have emerged independently in several geographical, scholarly and medical contexts. Also, the discussion about networks is not centred around just one or a few journals. However, the topics of the more frequently occurring journals are situated around the border between management and healthcare. Many individual examples of network configurations in specific care contexts are published in journals focused on specific medical fields. Hence, authors can be recommended to consider both specific medical journals and healthcare management journals, and as both types of journals are represented the references from more specific medical journals might serve as source material for healthcare practitioners.

This review shows that the concept of networks in healthcare can have several different meanings in practice. To allow for meta-learnings and analyses, scholars would be advised to provide clear descriptions of the organisational design of studied networks and preferably relate empirical descriptions to established classifications, such as modes of networks (Provan and Kenis, 2008), network centralisation (Brewster *et al.*, 2019; Huang and Provan, 2007; Provan *et al.*, 2009), and network initiators (Sampson *et al.*, 2015). The more elaborate empirical descriptions suggested would also enable future research focusing more on the impact of contextual factors.

The review also demonstrates that while network configurations have been applied in numerous cases, relatively few studies have presented outcome data. Networks are generally assumed to improve care, but some articles caution that networks may not always be better than alternative organisational configurations (Lorant *et al.*, 2019; Moore *et al.*, 2007; Sampson *et al.*, 2015). Hence, there is a need for more studies of what types of networks suit what purposes and contexts, as well as more research studying the effects of the network configuration in relation to the most common rationales for their use: efficiency and quality of care. This also has practical implications, pointing to the need to explicitly consider the main goals of the network and while designing the network also plan for measurements to follow up and evaluate goal fulfilment.

## Conclusions

Network configurations have been described in the scholarly literature over the last 3 decades with increasing frequency and primarily with a focus on long-term care for multimorbidity or chronic conditions, often in a context of mental healthcare or elderly care. Three clusters of articles were found corresponding to different conceptualisations and approaches to the use of networks in healthcare: *efficiency-enhancing cooperation*, *efficiency-enhancing integration*, and *involvement for cocreation*. In summary, it is clear from the descriptions of network configurations in the reviewed literature that the concept can be realised in various ways. This variety opens for several paths towards care delivered in a network. The three clusters point to different focuses and can thus be chosen to fit the key purposes of the network design – be it an urgent need to enhance efficiency or a need to strengthen co-creation of care.

## Appendix Search strategies

### Scopus:

((TITLE-ABS-KEY (health OR “health care” OR healthcare OR hospitals OR hospital)) AND (TITLE-ABS-KEY (organization OR organizations OR organisation OR organisations OR organised OR organized OR organizing OR organising OR organizational OR organizational OR administrat*OR management*)) AND ((TITLE-ABS-KEY (network* AND (co-creation OR co-production OR “value creation”))) OR (TITLE-ABS-KEY ((“Resource integration”) OR (“Integration resources”) OR (“service network”) OR (“value network”))))) AND NOT (INDEX (medline)) AND (LIMIT-TO (LANGUAGE, “English”))

### PubMed:

Search (((“health”[MeSH Terms] OR “health”[tiab] OR “delivery of health care”[MeSH Terms] OR “healthcare”[tiab] OR “health care”[tiab] OR “hospitals”[MeSH Terms] OR hospital[tiab] OR hospitals[tiab]) AND ((Network[tiab] AND (co-creation[tiab] OR co-production[tiab] OR “value creation”[tiab] OR “value co-creation”[tiab])) OR (“Resource integration”[tiab] OR “service network”[tiab] OR “value network”[tiab]))) AND (organization*[tiab] OR organisation*[tiab] OR organized[tiab] OR organized[tiab] OR organizing[tiab] OR organizing[tiab] OR administ*[tiab] OR management*[tiab] OR “Models, Organizational”[Mesh] OR “Organization and Administration”[Mesh:noexp] OR “Health Facility Administration”[Mesh:noexp] OR “Hospital Administration”[Mesh:noexp]) AND (English[lang]))
